# Regional electroencephalogram (EEG) alpha power and asymmetry in older adults: a study of short-term test–retest reliability

**DOI:** 10.3389/fnagi.2015.00177

**Published:** 2015-09-16

**Authors:** Karen J. Mathewson, Ali Hashemi, Bruce Sheng, Allison B. Sekuler, Patrick J. Bennett, Louis A. Schmidt

**Affiliations:** Department of Psychology, Neuroscience and Behaviour, McMaster UniversityHamilton, ON, Canada

**Keywords:** psychophysiology, aging, test–retest reliability, electroencephalogram (EEG), alpha power, frontal asymmetry

## Abstract

Although regional alpha power and asymmetry measures have been widely used as indices of individual differences in emotional processing and affective style in younger populations, there have been relatively few studies that have examined these measures in older adults. Here, we examined the short-term test–retest reliability of resting regional alpha power (7.5–12.5 Hz) and asymmetry in a sample of 38 active, community-dwelling older adults (*M* age = 71.2, *SD* = 6.5 years). Resting electroencephalogram recordings were made before and after a perceptual computer task. Pearson and intra-class correlations indicated acceptable test–retest reliability for alpha power and asymmetry measures in all regions. Interestingly, alpha asymmetry appeared to be less affected by the task than was alpha power. Findings suggest that alpha asymmetry may reflect more enduring, “trait-like” characteristics, while alpha power may reflect more “state-like” processes in older adults.

## Introduction

During relaxed wakefulness, the human electroencephalogram (EEG) is dominated by oscillations in the alpha frequency band (∼7.5–12.5 Hz). Resting alpha activity is reported to be unique to the individual (Benz et al., 2013), heritable (Smit et al., 2005; Anokhin et al., 2006), and stable (Salinsky et al., 1991). In certain conditions, however, individual differences in resting alpha activity reflect internal changes such as increasing fatigue (Simon et al., 2011), or reduced anxiety (Boutcher and Landers, 1988; Crabbe and Dishman, 2004), for instance, following the performance of demanding cognitive or physical tasks. Although these overall patterns are well-documented in younger adults, relatively little is known about alpha activity among healthy older adults.

Extant reports suggest that resting alpha activity is lower in older than younger adults (Sander et al., 2012), and further reduced in the presence of cognitive impairment (Koenig et al., 2005). Furthermore, the ability to modulate alpha power does not come easily to older adults (e.g., suppressing the processing of irrelevant information, Vaden et al., 2012; cf. Payne et al., 2013), and tends to break down readily under high-load conditions (Sander et al., 2012). Resting alpha activity appears to be both vulnerable to increased age and sensitive to the demands of effortful cognitive processing and physical activity.

Other studies have used the pattern of resting *frontal* EEG alpha asymmetry to understand dispositional mood and affective processing (e.g., Sutton and Davidson, 1997; Coan and Allen, 2004). For example, relatively greater frontal activity in the left hemisphere has been associated with behavioral approach and positive affect, whereas greater right-sided activity has been associated with behavioral inhibition and negative affect (Davidson, 1992, 2000; Sutton and Davidson, 1997). Frontal EEG asymmetry at rest has also been characterized as a diathesis that may be modified by salient stimuli of sufficient intensity (Petruzzello et al., 2001). For example, frontal alpha asymmetry is susceptible to procedures such as emotion induction via the use of emotional film clips (e.g., Wheeler et al., 1993) or specific training (e.g., Davidson et al., 2003), in younger adults. Frontal asymmetry also appears to be responsive to interventions such as mindfulness mediation training (Davidson et al., 2003), and cognitive behavior therapy (CBT; Moscovitch et al., 2011), which have been reported to sustain (Moynihan et al., 2013) or even increase left frontal asymmetry (Davidson et al., 2003).

Although studied far less frequently in older adults, relatively greater left frontal activity in this age group has been associated with facets of well-being, including life-satisfaction, autonomy, and engagement (Urry et al., 2004). In younger adults, such elements of well-being have been related to behavioral approach tendencies, sociability, and positive affect (e.g., Schmidt, 1999).

Although acceptable test–retest reliability of these measures has been demonstrated across different contexts (Schmidt et al., 2003), in younger adults (Tomarken et al., 1992; McEvoy et al., 2000; Winegust et al., 2014), children (Vuga et al., 2008), and in some clinical populations (e.g., Allen et al., 2004; Vuga et al., 2006; Schmidt et al., 2012), relatively few studies have examined short-term test–retest reliability of regional EEG alpha power and asymmetry measures in older individuals. If relatively greater left frontal asymmetry at rest is a reliable measure of psychological (Urry et al., 2004) and physiological (Davidson et al., 2003) well-being in older adults, then these metrics should show acceptable levels of test–retest reliability within the individuals tested. Examining the test–retest reliability of alpha measures is a first step in ensuring their psychometric soundness in older adults.

### The Present Study

Here, we assessed the short-term test–retest reliability of resting regional EEG alpha power and asymmetry measures in a community-dwelling sample of older adults, with particular attention to brain activity in the frontal regions. We examined resting regional alpha power and relative asymmetry before and after a challenging perceptual task. At the level of individual differences, moderate to strong correlations were expected between pre- and post-task resting conditions for both alpha power and asymmetry. Given that resting frontal asymmetry is described as dispositional (in the absence of intentional mood induction or interventions), asymmetry in frontal regions was expected to be correlated (i.e., reliable) between pre- and post-task conditions. Resting alpha power was expected to be more easily altered by the intervening task. As alpha power may increase due to fatigue, relaxation, or reduced anxiety following completion of a challenging task (Crabbe and Dishman, 2004), we anticipated that post-task levels of resting alpha power would be higher than pre-task levels.

## Materials and Methods

### Participants

Forty-one (20 females) older adults (*M* = 71.5 years, *SD* = 6.5 years, range: 61–86 years) were tested in the Vision and Cognitive Neuroscience Laboratory at McMaster University. All participants reported normal health, being free of neurological or psychiatric disorders, and living in the local community. Data from three participants were excluded because they did not have sufficient segments in either the pre- or post-task EEG recordings (<40, Towers and Allen, 2009), leaving data from 38 participants available for analysis. Sample characteristics are presented in **Table 1**.

**Table 1:** Sample characteristics.

	Women (*n* = 19)		Men (*n* = 19)	
	Mean *(SD)*	Range	Mean *(SD)*	Range
Age in years	70.6 (7.0)	25	71.9 (6.2)	20
Education in years	15.2 (3.2)	12	14.7 (2.8)	9
Handedness Score	7.6 (0.7)	2	7.4 (1.7)	7
Medication use *n* (%)	13 (68%)		12 (63%)	

*(a) A score of 8 on the handedness inventory (scale 0–8) indicates consistent right handedness, whereas a score of 0 indicates consistent left-handedness.*

*(b) Medication use represents the proportion of the sample taking prescribed medications.*

### Procedures

Participants were introduced to the laboratory at McMaster University and briefed about the study procedures. Informed consent was obtained prior to testing. Throughout the testing session, participants were seated in a comfortable chair in a dimly-lit, copper-shielded room maintained at a comfortable temperature. Regional EEG data were continuously recorded during seated rest, prior to performance of a visual perception task (T1), and immediately following the task (T2), as part of a larger ERP study. Following the EEG testing, resting blood pressure and a brief cardiac recording were taken, after which participants completed several questionnaires for use in the larger study. Upon completion of testing, participants were debriefed and given a nominal reimbursement for their time and travel expenses. The study received clearance from the McMaster Research Ethics Board.

### Regional EEG Data Collection and Reduction

#### EEG Recording

Resting EEG data were recorded continuously using a 256-channel HydroCel Geodesic Sensor Net [Electrical Geodesics, Inc., Eugene, OR, USA (EGI)] during a 6-min baseline before and after the visual perceptual task, alternating 1-min intervals between eyes-closed (EC) and eyes-open (EO) conditions. During acquisition, impedances were kept below 50 kΩ, in accord with recommendations in the Electrical Geodesics, Inc. (2006) published by EGI. EEG signals were sampled at 250 Hz, referenced to the vertex (Cz), digitized with a 16-bit analog-to-digital converter (ADC), and hardware-filtered using an analog filter from 0.01 to 100 Hz. Participants were instructed to relax and minimize movements.

#### EEG Data Reduction

Oﬄine, any channel with consistent artifact was interpolated from the channel’s nearest neighboring sites prior to further analysis using Brain Vision Analyzer 2.0.4 (Brain Products, GmbH, Gilching, Germany). EEG data were band-pass filtered between 0.1 and 50 Hz, the sampling rate was changed from 250 to 256 Hz, noisy channels were interpolated, and the data were edited for artifacts, using a ±200 μV criterion. If artifacts were present in one channel, data in all channels were excluded for that epoch. Artifact-free EEG data from the EC and EO conditions were analyzed separately using a fast Fourier transform (FFT), with a Hanning window of 1-s width with 10% overlap of epochs. EEG power was derived for the traditional frequency bands: delta, 0.5–3.5 Hz; theta, 3.5–7.5 Hz; alpha, 7.5–12.5 Hz; beta 12.5–30 Hz; and gamma, 30–50 Hz. Given previously reported associations between frontal alpha asymmetry and affective processing, power in the alpha band was of particular interest. For each 1-min interval, EEG data were analyzed beginning 5-s after the instruction to open or close the eyes. Estimates of EEG power were based on an average of 229 (*SD* = 62) 1-s epochs, with a minimum of 56 epochs, and averaged within the EC or EO conditions separately.

### EEG Alpha Measures

#### Regional Alpha Power

Electroencephalogram rhythms may be measured in terms of power (μV^2^) or its square root, amplitude (μV). EEG power was derived for all frequency bands using amplitude (μV). However, we refer to “alpha power” from this point forward, as this term is more commonly understood. EEG clusters of electrode channels were identified as corresponding to each of the relevant sites from the International 10/20 Electrode Placement System (Jasper, 1958). EEG signals for each condition (pre- vs. post-task; EC vs. EO) were averaged separately to form local power values in the left hemisphere for Fp1 (sites 27, 32, 33, 34, 37), F3 (sites 36, 40, 41, 42, 49, 50), F7 (39, 46, 47, 48, 54), C3 (sites 51, 52, 58, 59, 60, 65, 66), T3 (sites 63, 68, 69, 70, 74), P3 (sites 76, 77, 85, 86, 87, 97, 98), P5 (sites 84, 94, 95, 96, 105), O1 (sites 116, 123, 124, 125, 136), and eight homologous sites in the right hemisphere (Fp2, F4, F8, C4, T4, P4, P6, and O2). EEG power values for each (clustered) site were natural-log (ln) transformed to normalize the data, and reported separately for the EC and EO conditions.

#### Regional Alpha Asymmetry

Eight measures of regional alpha asymmetry (prefrontal, mid-frontal, lateral frontal, central, temporal, parieto-temporal, parietal, occipital regions) were calculated separately by subtracting natural-log transformed regional EEG alpha power in the left hemisphere from natural-log transformed power at homologous sites in the right hemisphere [e.g., ln(right) power minus ln(left) power], separately for the EC and EO conditions, during pre- and post-task rest. EEG alpha power is inversely related to cortical activity. Therefore, positive values of frontal asymmetry reflect relatively greater alpha power in the right hemisphere, indicating greater activity in the left hemisphere, whereas negative values represent relatively greater alpha power in the left hemisphere, indicating greater activity in the right hemisphere (Davidson, 1992).

## Results

### Test–Retest Reliability of Regional Alpha Power and Asymmetry Measures Pre- vs. Post-Task

Mean values for pre-task (T1) and post-task (T2) alpha power and asymmetry are presented by region for the EC and EO conditions in **Tables 2A,B**, respectively.

**Table 2:** Mean (SD) and test–retest reliability coefficients for left and right resting EEG alpha power (in *μ*V) and regional asymmetry in older adults, before and after task performance for **(A)** eyes closed and **(B)** eyes open conditions (*n* = 38).

Measure	Time 1 Mean (*SD*) Pre-task	Time 2 Mean (*SD*) Post-task	T1 to T2 change *t*-value	Pearson *r* correlation	Intraclass correlation
**(A) Eyes closed (EC)**					
**Regional power (site)**					
Fp1	1.34 (1.0)	1.52 (1.0)	3.51^∗∗^	0.95^∗∗∗^	0.95^∗∗∗^
F3	0.93 (1.0)	1.15 (1.0)	4.36^∗∗∗^	0.96^∗∗∗^	0.95^∗∗∗^
F7	1.20 (0.9)	1.35 (0.9)	3.20^∗∗^	0.95^∗∗∗^	0.95^∗∗∗^
C3	0.71 (1.0)	0.96 (0.9)	4.84^∗∗∗^	0.96^∗∗∗^	0.95^∗∗∗^
T3	0.79 (0.9)	1.03 (0.8)	5.15^∗∗∗^	0.95^∗∗∗^	0.94^∗∗∗^
P3	1.10 (1.1)	1.38 (1.0)	6.25^∗∗∗^	0.97^∗∗∗^	0.97^∗∗∗^
P5	1.25 (1.0)	1.50 (0.9)	5.04^∗∗∗^	0.95^∗∗∗^	0.95^∗∗∗^
O1	1.31 (1.1)	1.55 (0.9)	3.26^∗∗^	0.91^∗∗∗^	0.90^∗∗∗^
Fp2	1.38 (1.0)	1.52 (1.0)	2.71^∗^	0.95^∗∗∗^	0.95^∗∗∗^
F4	1.08 (1.0)	1.25 (0.9)	3.05^∗∗^	0.94^∗∗∗^	0.93^∗∗∗^
F8	1.28 (0.9)	1.42 (0.8)	2.88^∗∗^	0.94^∗∗∗^	0.94^∗∗∗^
C4	0.66 (0.9)	0.92 (0.8)	6.88^∗∗∗^	0.97^∗∗∗^	0.97^∗∗∗^
T4	0.84 (0.8)	1.05 (0.8)	4.35^∗∗∗^	0.94^∗∗∗^	0.93^∗∗∗^
P4	1.04 (1.1)	1.34 (1.0)	5.05^∗∗∗^	0.95^∗∗∗^	0.94^∗∗∗^
P6	1.18 (1.1)	1.47 (1.0)	5.33^∗∗∗^	0.95^∗∗∗^	0.95^∗∗∗^
O2	1.30 (1.2)	1.54 (1.0)	3.09^∗∗^	0.92^∗∗∗^	0.91^∗∗∗^
**Regional asymmetry**					
Fp2-Fp1	0.04 (0.13)	<0.01 (0.10)	2.44^∗^	0.68^∗∗∗^	0.65^∗∗∗^
F4-F3	0.15 (0.35)	0.11 (0.34)	1.41	0.84^∗∗∗^	0.84^∗∗∗^
F8-F7	0.08 (0.26)	0.07 (0.27)	0.34	0.76^∗∗∗^	0.76^∗∗∗^
C4-C3	-0.05 (0.47)	-0.04 (0.30)	0.20	0.82^∗∗∗^	0.75^∗∗∗^
T4-T3	0.06 (0.34)	0.02 (0.33)	1.17	0.85^∗∗∗^	0.85^∗∗∗^
P4-P3	-0.07 (0.41)	-0.04 (0.41)	1.01	0.91^∗∗∗^	0.91^∗∗∗^
P6-P5	-0.07 (0.46)	-0.02 (0.44)	1.34	0.87^∗∗∗^	0.87^∗∗∗^
O2-O1	-0.01 (0.26)	-0.02 (0.30)	0.24	0.91^∗∗∗^	0.90^∗∗∗^
**(B) Eyes open (EO)**					
**Regional power (site)**					
Fp1	0.82 (0.7)	0.93 (0.7)	2.27^∗^	0.90^∗∗∗^	0.90^∗∗∗^
F3	0.24 (0.8)	0.42 (0.8)	3.45 ^∗∗^	0.92^∗∗∗^	0.92^∗∗∗^
F7	0.65 (0.7)	0.77 (0.7)	2.75^∗∗^	0.93^∗∗∗^	0.93^∗∗∗^
C3	0.12 (0.9)	0.33 (0.9)	3.09^∗∗^	0.90^∗∗∗^	0.89^∗∗∗^
T3	0.36 (0.8)	0.62 (0.8)	4.80^∗∗∗^	0.92^∗∗∗^	0.91^∗∗∗^
P3	0.27 (0.9)	0.55 (0.9)	5.26^∗∗∗^	0.93^∗∗∗^	0.93^∗∗∗^
P5	0.41 (0.9)	0.66 (0.8)	4.76^∗∗∗^	0.92^∗∗∗^	0.92^∗∗∗^
O1	0.25 (0.9)	0.48 (0.7)	3.07^∗∗^	0.85^∗∗∗^	0.84^∗∗∗^
Fp2	0.86 (0.7)	0.95 (0.7)	1.49	0.86^∗∗∗^	0.86^∗∗∗^
F4	0.39 (0.8)	0.57 (0.7)	2.54^∗^	0.86^∗∗∗^	0.84^∗∗∗^
F8	0.70 (0.7)	0.89 (0.6)	3.23^∗∗^	0.88^∗∗∗^	0.87^∗∗∗^
C4	0.08 (0.8)	0.31 (0.8)	5.63^∗∗∗^	0.95^∗∗∗^	0.95^∗∗∗^
T4	0.40 (0.8)	0.63 (0.7)	3.92^∗∗∗^	0.90^∗∗∗^	0.89^∗∗∗^
P4	0.19 (0.9)	0.47 (0.9)	4.32^∗∗∗^	0.90^∗∗∗^	0.90^∗∗∗^
P6	0.31 (0.9)	0.58 (0.8)	4.41^∗∗∗^	0.90^∗∗∗^	0.90^∗∗∗^
O2	0.19 (0.9)	0.42 (0.8)	3.08^∗∗^	0.86^∗∗∗^	0.85^∗∗∗^
**Regional asymmetry**					
Fp2-Fp1	0.04 (0.19)	0.02 (0.16)	0.93	0.54^∗∗∗^	0.53^∗∗∗^
F4-F3	0.15 (0.29)	0.14 (0.32)	0.18	0.82^∗∗∗^	0.81^∗∗∗^
F8-F7	0.06 (0.25)	0.12 (0.28)	1.57	0.58^∗∗∗^	0.57^∗∗∗^
C4-C3	-0.05 (0.50)	-0.02 (0.36)	0.57	0.80^∗∗∗^	0.76^∗∗∗^
T4-T3	0.04 (0.36)	<0.01 (0.37)	0.93	0.76^∗∗∗^	0.76^∗∗∗^
P4-P3	-0.08 (0.25)	-0.08 (0.31)	0.02	0.75^∗∗∗^	0.74^∗∗∗^
P6-P5	-0.10 (0.33)	-0.08 (0.29)	0.53	0.72^∗∗∗^	0.72^∗∗∗^
O2-O1	-0.06 (0.24)	-0.06 (0.24)	0.41	0.86^∗∗∗^	0.86^∗∗∗^

*^∗^p < 0.05, ^∗∗^p < 0.01, ^∗∗∗^p < 0.001.*

#### Regional Alpha Power

Test–retest reliability between pre-task and post-task alpha power measures was excellent for the EC and EO conditions (EC: ICCs = 0.90–0.97, *p*s < 0.001; EO: ICCs = 0.84–0.95, *p*s < 0.001). Importantly, measures of pre- and post-task alpha power were also highly correlated, indicating that individual differences in alpha power evident before the task (and their rank order) were clearly preserved after the task (all *p*s < 0.001; see **Table 2**).

#### Regional Alpha Asymmetry

**FIGURE 1:**
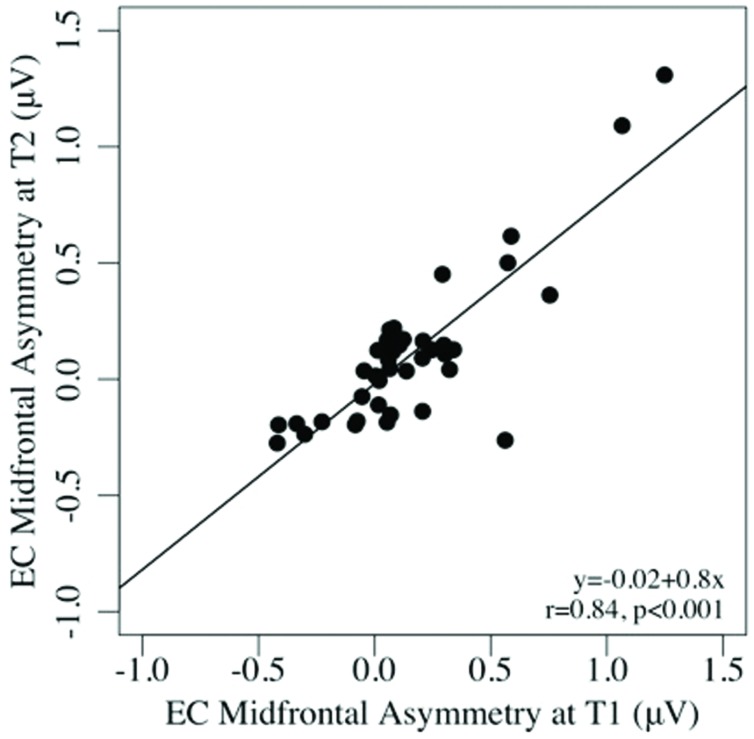
**Scatterplots of the associations between mid-frontal asymmetry in the eyes-closed, pre-task (T1), and post-task (T2) conditions**.

**FIGURE 2:**
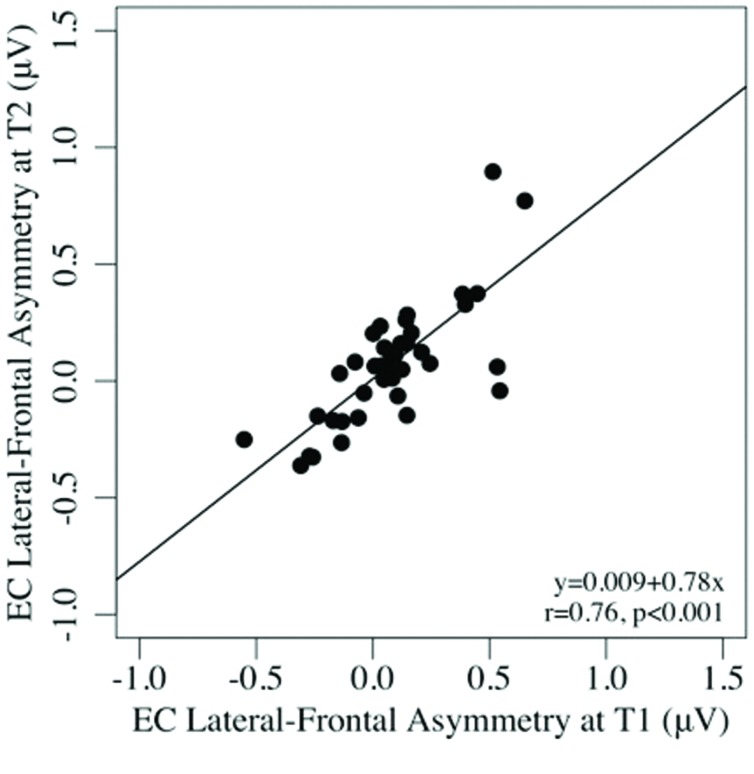
**Scatterplots of the associations between lateral-frontal asymmetry in the eyes-closed, pre-task (T1), and post-task (T2) conditions**.

Test–retest reliability between pre- and post-task regional asymmetry was very good (EC: ICCs = 0.65–0.91, *p*s < 0.001; EO: ICCs = 0.53–0.86, *p*s < 0.001). As well, pre- and post-task asymmetry values were highly correlated for both the EC and EO conditions for all regions tested (all *p*s < 0.001; see **Table 2**). Individual differences in frontal alpha asymmetry (and their rank order) seen at T1 were well-preserved at T2, after the task (see **Figures 1** and **2**).

### Analysis of Resting EEG Power: Eyes Closed Condition

**FIGURE 3:**
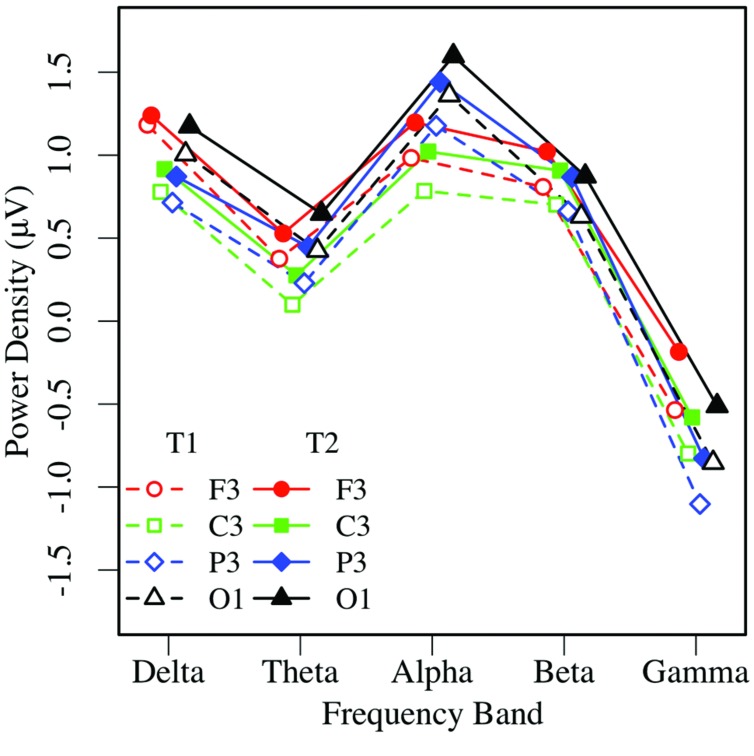
**Eyes-closed EEG power in the left hemisphere, by frequency, region, and condition (T1 vs. T2)**.

**FIGURE 4:**
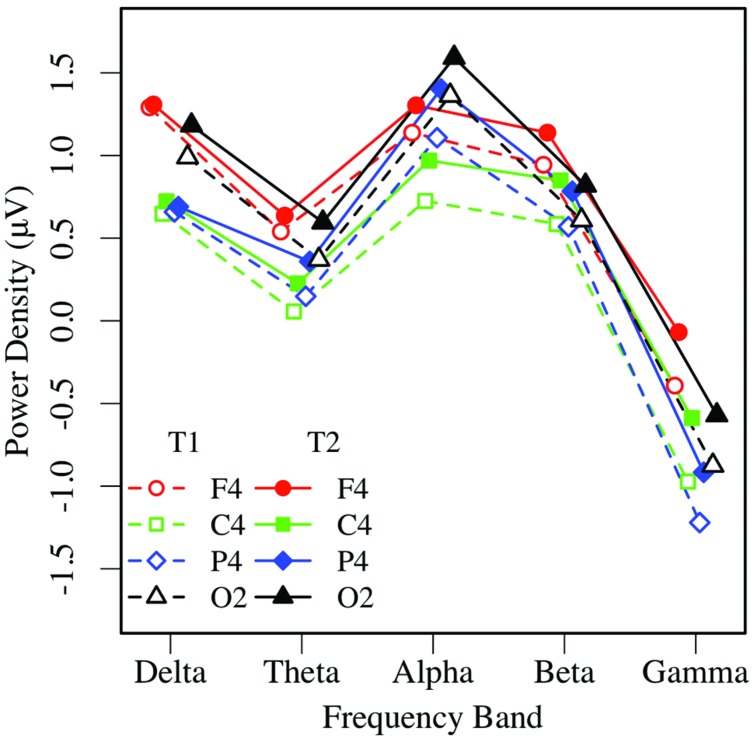
**Eyes-closed EEG power in the right hemisphere, by frequency, region, and condition (T1 vs. T2)**.

Eyes-closed pre- and post-task resting EEG activity was analyzed in a 2 × 5 × 2 × 4 omnibus ANOVA, with measurement occasion (pre-task, T1, vs. post-task, T2), frequency (delta, theta, alpha, beta, gamma), hemisphere (left, right), and region (mid-frontal, central, parietal, occipital) as factors. Main effects of measurement occasion, frequency, and region (*p*s < 0.001) were qualified by two-way interactions. Frequency interacted with measurement occasion, *F*(4,148) = 3.32, *p* < 0.03, $\eta_{p}^{2}$ = 0.08, and region *F*(12,444) = 16.97, *p* < 0.001, $\eta_{p}^{2}$ = 0.31, and the regional effect interacted with hemisphere *F*(3,111) = 4.04, *p* < 0.02, $\eta_{p}^{2}$ = 0.10, with no other effects or interactions, *p*s > 0.12. Unadjusted pairwise tests indicated that EEG power was greater in the post-task (T2: *M* = 0.58 μV, *SE* = 0.09) than pre-task condition (T1: *M* = 0.35 μV, *SE* = 0.11; see **Figures 3** and **4**), and greater at the alpha frequency (*M* = 1.14 μV, *SE* = 0.15) than all other frequencies (*p*s < 0.01), except delta (*M* = 0.92 μV, *SE* = 0.09), *p >* 0.10. EEG power was also greater in mid-frontal (*M* = 0.66 μV, *SE* = 0.11) than central (*M* = 0.31 μV, *SE* = 0.10), *p* < 0.001, parietal (*M* = 0.33 μV, *SE* = 0.10), *p* < 0.001, or occipital regions (*M* = 0.56 μV, *SE* = 0.10), *p <* 0.06. In sum, EC resting EEG power differed by frequency, and where and when it was measured, with a pattern that suggested mid-frontal asymmetry in the alpha band frequency.

Because mid-frontal alpha asymmetry was of particular interest to this study, a 2 × 2 (measurement occasion, hemisphere) ANOVA of alpha power was performed for the mid-frontal region. Mid-frontal alpha power was greater in the post-task (T2: *M* = 1.20 μV, *SE* = 0.15) than the pre-task condition (T1: *M* = 1.00 μV, *SE* = 0.16), *F*(1,37) = 14.62, *p* < 0.001, $\eta_{p}^{2}$ = 0.28, and significantly greater in the right hemisphere (*M* = 1.16 μV, *SE* = 0.15) than the left (*M* = 1.04 μV, *SE* = 0.16), *F*(1,37) = 5.61, *p* < 0.03, $\eta_{p}^{2}$ = 0.13, with no interaction, *p*s > 0.16. Relatively greater frontal alpha power in the right hemisphere reflected greater left frontal asymmetry (i.e., more activity in the left frontal region) in the EC condition.

### Analysis of Resting EEG Power: Eyes-Open Condition

**FIGURE 5:**
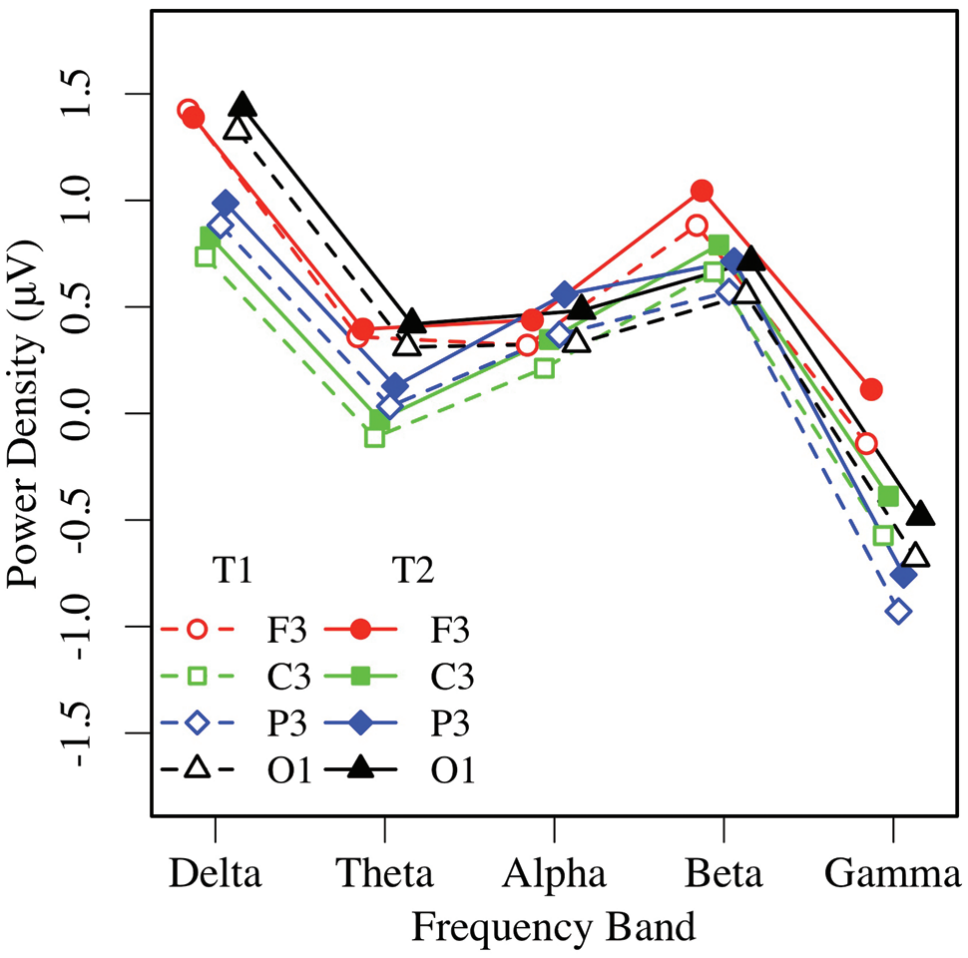
**Eyes-open EEG power in the left hemisphere by frequency, region, and condition (T1 vs. T2)**.

**FIGURE 6:**
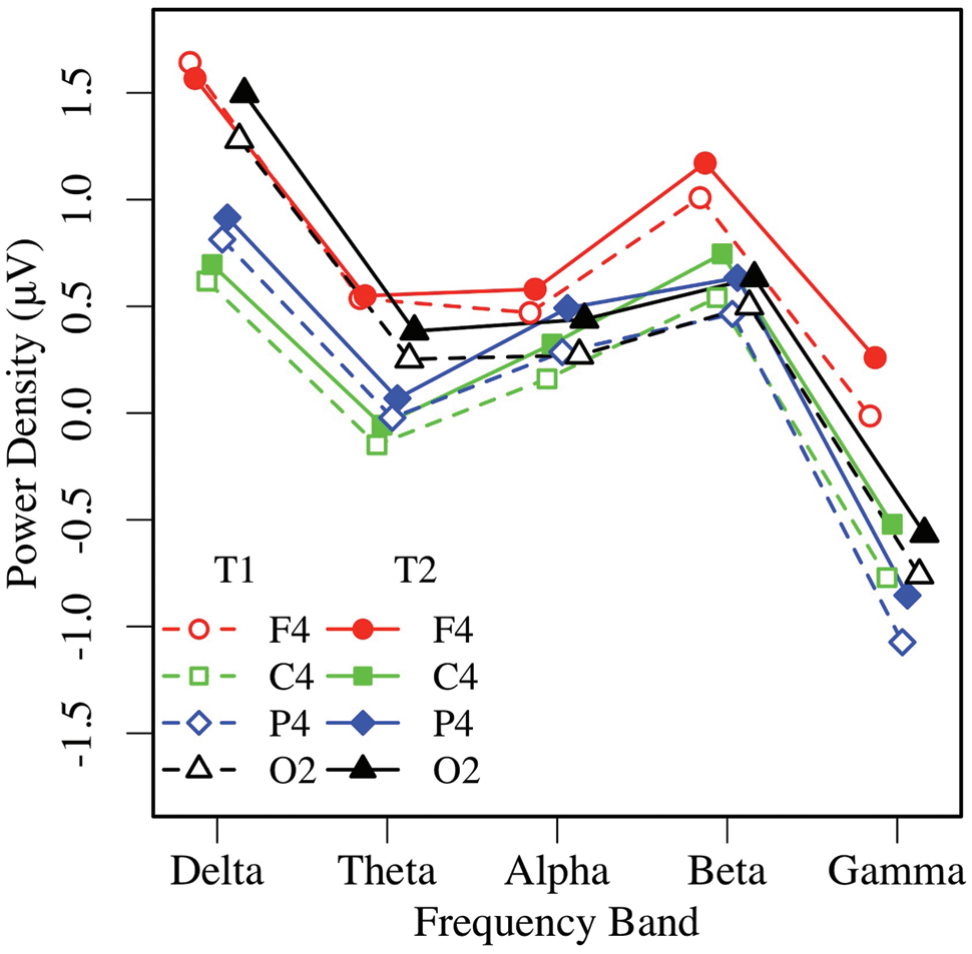
**Eyes-open EEG power in the right hemisphere by frequency, region, and condition (T1 vs. T2)**.

Similar to the EC condition, an omnibus 2 × 5 × 2 × 4 ANOVA of EO resting EEG power yielded main effects of measurement occasion, frequency, and region (*p*s < 0.01), and significant region by frequency, *F*(12,444) = 23.66, *p* < 0.001, $\eta_{p}^{2}$ = 0.39, and region by hemisphere, *F*(3,111) = 4.53, *p* < 0.02, $\eta_{p}^{2}$ = 0.11 interactions. Post-task EO EEG power was higher (T2: *M* = 0.43 μV, *SE* = 0.07), than pre-task power (T1: *M* = 0.25 μV, *SE* = 0.09; see **Figures 5** and **6**). Resting EO power was greater in the delta frequency band (*M* = 1.13 μV, *SE* = 0.09) than the other frequencies, *p*s < 0.001, and greater in mid-frontal (*M* = 0.66 μV, *SE* = 0.09), than the other regions, *p*s < 0.001, with no other effects or interactions, *p*s > 0.13. Like the EC condition, EO resting EEG power differed by frequency, and where and when it was measured, and exhibited a pattern that suggested significant asymmetry in mid-frontal alpha power.

To ascertain whether asymmetry was present in the EO condition, a 2 × 2 ANOVA of EO mid-frontal alpha power was performed, showing that mid-frontal alpha was greater in the post-task (T2: *M* = 0.50 μV, *SE* = 0.12) than pre-task condition (T1: *M* = 0.32 μV, *SE* = 0.13), *F*(1,37) = 8.96, *p* < 0.01, $\eta_{p}^{2}$ = 0.20, and significantly greater in the right hemisphere (*M* = 0.48 μV, *SE* = 0.12) than the left (*M* = 0.33 μV, *SE* = 0.13), *F*(1,37) = 9.52, *p <* 0.01, $\eta_{p}^{2}$ = 0.21, with no interaction, *p* > 0.85. Similarly to the EC condition, relatively greater EO alpha activity in the right hemisphere reflected greater left frontal asymmetry (more activity in the left frontal region), across the group.

Overall, resting EC and EO alpha power increased significantly following task performance in all regions tested^1^, and mid-frontal alpha power was relatively greater in the right hemisphere, reflecting left frontal asymmetry in both EC and EO conditions.

#### Analysis of Resting Alpha Asymmetry

**FIGURE 7:**
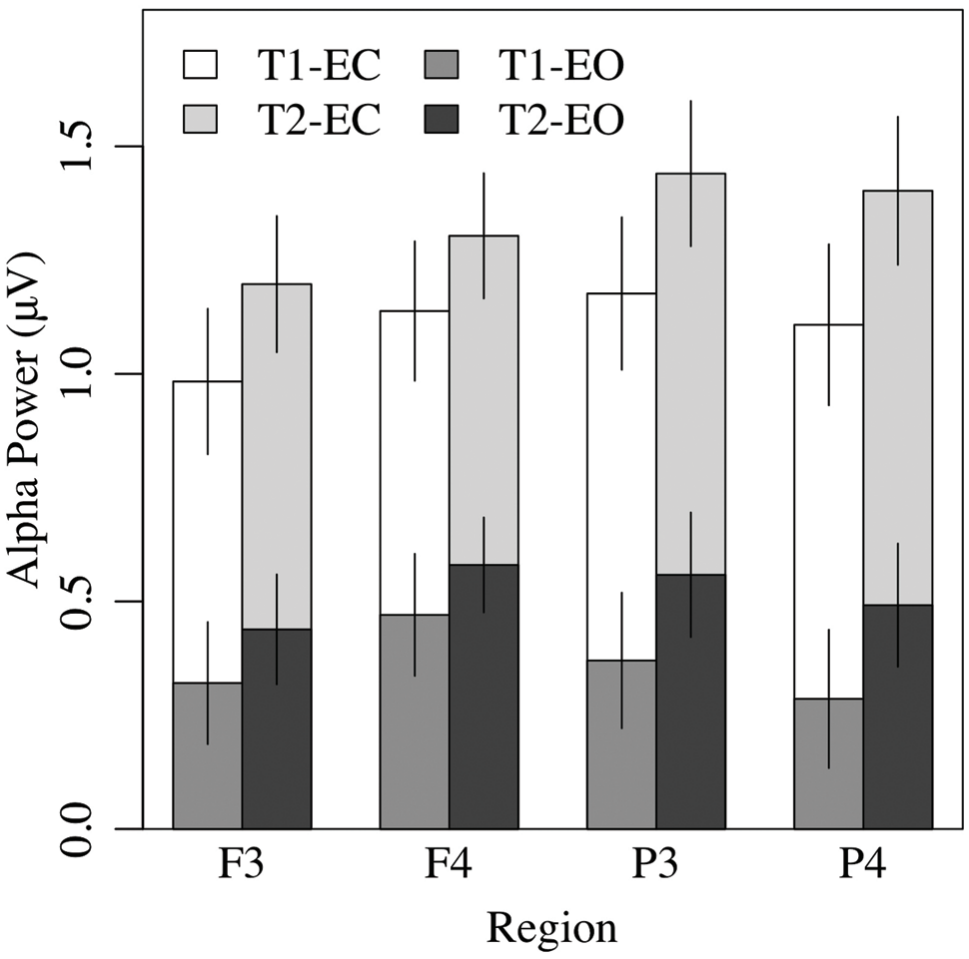
**Overall EEG alpha power in the eyes-closed and eyes-open conditions was greater at T2 than T1, and greater in the frontal right hemisphere than the left**.

Alpha asymmetry is most commonly analyzed at frontal (prefrontal, mid-frontal, lateral-frontal), and some posterior (e.g., parietal) sites. Therefore, measures of resting alpha asymmetry from six regions (prefrontal, mid-frontal, lateral-frontal, central, parietal, occipital) were selected and submitted to a 2 × 2 × 6 (EC vs. EO condition by measurement occasion by region) ANOVA. The analysis revealed only a main effect of region, *F*(5,185) = 3.20, *p* < 0.04, $\eta_{p}^{2}$ = 0.08, with no other effects or interactions, *p* > 0.15. Pairwise tests indicated that mid-frontal asymmetry (*M* = 0.14 μV, *SE* = 0.05) was greater than alpha asymmetry at all other sites (all *p*s < 0.03, except the lateral frontal region (*M* = 0.08 μV, *SE* = 0.04), *p* < 0.07; see **Figure 7**). The magnitude of parietal asymmetry (*M* = -0.07 μV, *SE* = 0.05) did not differ from than that of central (*M* = -0.04 μV, *SE* = 0.06), or occipital asymmetry (*M* = -0.04 μV, *SE* = 0.04), *p*s > 0.50, but was significantly lower than that of lateral frontal asymmetry, *p* < 0.05. To test whether any of the asymmetry values differed significantly from zero, regional asymmetry values at each site were collapsed across the EC and EO conditions and entered in one-sample *t*-tests. Only mid-frontal asymmetry at T1 and T2, *p*s < 0.03, and lateral-frontal asymmetry at T2, *p* < 0.04, differed significantly from zero (all other *p*s > 0.09).

#### Predictors of Pre- to Post-Task Increases in Alpha Activity

Given that regional measures of resting alpha power were greatest during post-task rest, an additional set of analyses was performed to ascertain whether the increase was related to individual characteristics, namely, age, sex, education level, handedness, or medication status (taking prescribed medications vs. taking none).

A series of regression analyses was performed on the pre- to post-task change in EC alpha power at each of the 16 sites, with age, sex, education, and handedness as independent predictors. These analyses indicated that sex accounted for significant variance (12–31%) in the post-task increase in alpha power (difference scores) at virtually every site, *p*s < 0.05, with trends for T3 (*p* < 0.09, 9%) and O2 (*p* < 0.06, 11%). Age, education level, handedness, and medication status were non-significant at every site (all *p*s > 0.20).

Similar results were obtained for EO alpha power. Sex explained significant variance (11–28%) in the post-task increase in alpha power at almost every site, *p*s < 0.05, with trends for Fp2 (*p* < 0.06, 10%) and C3 (*p* < 0.11, 8%). Age, education level, handedness, and medication status did not reach significance at any site (all *p*s > 0.06) except for the increase at P5, which was positively predicted by age (*p <* 0.05, 9%; see **Table 3**).

**Table 3:** Regression results for sex as a predictor of increased resting EC alpha power (*n* = 38).

	B	*SE*	*sr^2^*		B	*SE*	*sr^2^*
**L Hem**				**R Hem**			
**Site**				**Site**			

**EC**					
Fp1	0.26^∗^	0.10	0.16	Fp2	0.26^∗^	0.11	0.15
F3	0.25^∗^	0.10	0.16	F4	0.32^∗∗^	0.11	0.20
F7	0.21^∗^	0.10	0.12	F8	0.32^∗∗^	0.09	0.26
C3	0.25^∗^	0.10	0.16	C4	0.17^∗^	0.07	0.14
T3	0.17	0.10	0.09	T4	0.22^∗^	0.09	0.14
P3	0.31^∗∗^	0.08	0.31	P4	0.31^∗^	0.12	0.17
P5	0.30^∗∗^	0.09	0.24	P6	0.31^∗^	0.11	0.20
O1	0.34^∗^	0.15	0.13	O2	0.31^†^	0.16	0.11
**EO**					
Fp1	0.23^∗^	0.10	0.13	Fp2	0.23^†^	0.12	0.10
F3	0.23^∗^	0.11	0.13	F4	0.34^∗^	0.13	0.16
F7	0.19^∗^	0.09	0.11	F8	0.37^∗∗^	0.11	0.27
C3	0.23	0.14	0.08	C4	0.27^∗∗^	0.07	0.26
T3	0.23^∗^	0.11	0.11	T4	0.30^∗∗^	0.11	0.18
P3	0.34^∗∗^	0.10	0.26	P4	0.35^∗∗^	0.12	0.19
P5	0.30^∗∗^	0.10	0.20	P6	0.40^∗∗^	0.11	0.28
O1	0.38^∗^	0.14	0.17	O2	0.36^∗^	0.15	0.15

*^∗^p < 0.05, ^∗∗^p < 0.01, ^†^p < 0.06.*

*(a) Post-task increases in alpha power were larger in women than men.*

**FIGURE 8:**
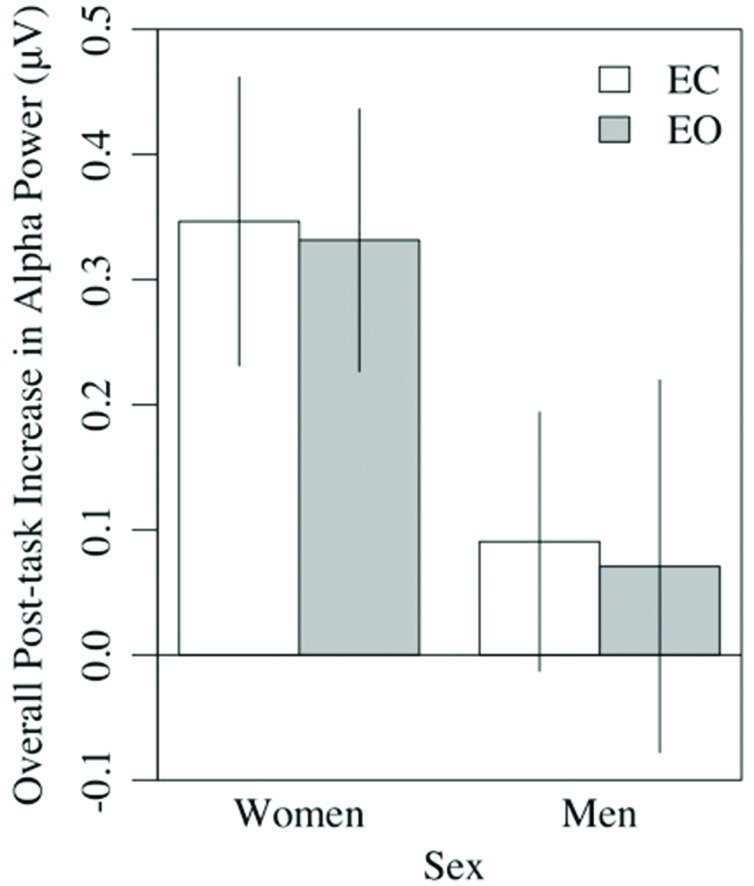
**Overall post-task increases in alpha power in the eyes-closed and eyes-open conditions were greater in women than men**.

A 2 × 8 (hemisphere × region) ANCOVA of the power difference scores, statistically controlled for sex, indicated that the post-task increase in EC alpha power was larger in women (*M* = 0.36, *SE* = 0.06) than men (*M* = 0.09, *SE* = 0.06), *F*(1,36) = 10.19, *p <* 0.01, $\eta_{p}^{2}$ = 0.22, and greater in parietal (P3, P4, P5, P6) than frontal regions (Fp1, Fp2, F3, F4, F7, F8), all *p*s < 0.01 (pairwise), *F*(7,252) = 4.19, *p* < 0.01, $\eta_{p}^{2}$ = 0.10 (see **Figure 8**). Numerically, the effect size for sex exceeded that of region. There were no interactions, *p*s > 0.30. The post-task increase in EO alpha power was similar, being larger for women (*M* = 0.35 μV, *SE* = 0.07) than men (*M* = 0.07 μV, *SE* = 0.07), *F*(1,36) = 8.49, *p* < 0.01, $\eta_{p}^{2}$ = 0.19, and greater in temporo-parietal (T3, T4, P3, P4, P5, P6), relative to frontal regions (Fp1, Fp2, F3, F4, F7, F8), all *p*s < 0.03 (pairwise), *F*(7,252) = 6.15, *p* < 0.001, $\eta_{p}^{2}$ = 0.15. For the EO condition, sex interacted with hemisphere, with women showing greater right-sided increases in alpha power relative to left-sided increases (R: *M* = 0.37 μV, *SE* = 0.07; L: *M* = 0.33 μV, *SE* = 0.07), and men showing the reverse (R: *M* = 0.06 μV, *SE* = 0.07; L: *M* = 0.08 μV, *SE* = 0.07). In contrast to the results with change scores, parallel analyses of simple pre-task (T1) resting alpha power revealed no sex differences for either the EC (*p* > 0.11) or EO (*p* > 0.24) conditions. We note, though, that resting alpha power was nominally higher in men (EC: *M* = 1.33 μV, *SE* = 0.22; EO: *M* = 0.54 μV, *SE* = 0.18) than women (EC: *M* = 0.84 μV, *SE* = 0.22; EO: *M* = 0.24 μV, *SE* = 0.18) at T1, prior to task performance.

## Discussion

Inherent to the notion that resting EEG alpha power and asymmetry are valid measures of dispositional and state processes is the assumption that within-individual differences in these measures remain stable across time and contexts. An initial step in addressing their validity is to confirm their reliability. Here, we sought to establish test–retest reliability in a sample of healthy older adults. We found that individual differences in resting regional EEG alpha power and asymmetry showed good-to-excellent test–retest reliability from pre- to post-task conditions at all sites tested. While similar test–retest reliability has been reported in non-clinical (e.g., Tomarken et al., 1992; McEvoy et al., 2000) and clinical (Schmidt et al., 2012) samples of younger adults, these findings appear to be the first to demonstrate short-term test–retest reliability of regional EEG power and asymmetry in a sample of healthy older adults using a dense array methodology.

The second major finding was that at virtually all of the individual sites tested, resting alpha power increased following performance of a perceptual task, for both the EC and EO conditions. The topography of the increase in alpha power indicated a global change in participants’ electrocortical resting state in response to the intervening task, similar to that reported by Simon et al. (2011) in drowsy drivers. We believe the increase in alpha power following the perceptual task reported here may be related to fatigue, similar to the increase in alpha power that occurs after prolonged driving, another visual perception task demanding attentional control and vigilance. In our sample, post-task alpha power increases were more substantial in older women than in older men, the latter of whom showed more incremental and more variable changes (**Figure 8**). Because alpha power during pre-task rest was non-significantly higher in men than women, the larger increase in older women served to equalize alpha power differences between sexes in the post-task condition.

The third finding was that because the increase in alpha power from pre- to post-task conditions was global, the balance between alpha power in left and right frontal regions did not change. Consistent with a large literature linking left frontal asymmetry to behavioral approach and positive affect in young adults, resting EEG signals in this sample of healthy older adults exhibited significantly greater resting left frontal than right frontal activation in both EC and EO conditions, at T1 and T2. In addition, the asymmetry pattern was localized primarily to mid-frontal sites, where asymmetry was substantially different from zero.

Although the literature on asymmetry in older adults is scant, our findings are in line with the evidence currently available. Greater left frontal asymmetry has been reported in healthy adults aged 57–60, where it was positively associated with well-being, approach behavior, and agency (Urry et al., 2004). Our findings are also consistent with a study in which left frontal asymmetry was maintained in adults 65 years or older who participated in 8 weeks of mindfulness meditation training, in contrast to a decline in matched wait-list controls over the same period (Moynihan et al., 2013). Increases in left frontal asymmetry, along with positive changes in immune function, have also been reported in young and middle-aged adults who participated in mindfulness mediation training (Davidson et al., 2003), and in socially anxious adults participating in CBT (e.g., Moscovitch et al., 2011).

Overall, our data suggest acceptable short-term test–retest reliability in alpha power and asymmetry at the level of the individual. The present results are in line with the extensive literature on frontal asymmetry in younger adults. These findings also suggest that while mean measures of frontal asymmetry did not change from pre-to post-task conditions, mean levels of alpha power were uniquely sensitive to the experimental task.

### Limitations

There are at least two limitations to the interpretation of the findings reported here. First, the findings were derived from a relatively healthy, small sample of community-dwelling older adults, which may limit their generalizability to older adults with significant health problems, and limited mobility, social connections, or economic resources. We note, though, that the adults in our sample spanned a wide range of ages (61–86 years), representing both “young-old” individuals who were still employed or only recently retired, as well as “old–old” adults who were well into their retirement years.

Second, while we have demonstrated sound test–retest reliability with respect to individual differences in resting alpha power and asymmetry in the context of task performance, we have not shown this reliability over an extended period of time. It would be important to establish comparable reliability of alpha power and frontal asymmetry in a longitudinal sample. Yet, we note that post-task increases in resting alpha power have only rarely been reported in the literature, and usually in the context of physical tasks.

## Conclusion

Resting frontal alpha power and asymmetry have been linked to stable individual differences in dispositional variables in many previous studies. Simultaneously, they may also demonstrate state-dependent variation in response to changing environmental demands. In the present study, individual differences in resting EEG alpha power and asymmetry from two occasions were highly reliable in a sample of older adults. Although mean levels of alpha power were uniquely sensitive to experimental context (and may have represented greater fatigue in older women than older men), mean measures of frontal alpha asymmetry did not change. Demonstrating within-subject reliability across contexts serves to validate the notion that these measures actually reflect meaningful individual differences that are of potential interest to aging and personality and emotion research. Although studies of EEG alpha power and asymmetry have long been used as psychophysiological measures in younger adults, the results of the present study documenting test–retest reliability of resting frontal EEG alpha power and asymmetry in older adults support the use of these psychophysiological measures in future studies of healthy aging.

## Conflict of Interest Statement

The authors declare that the research was conducted in the absence of any commercial or financial relationships that could be construed as a potential conflict of interest.

## Footnotes

1 Similar to findings reviewed by Crabbe and Dishman (2004), post-task increases in absolute alpha power were not reflected in analyses of relative alpha power. When EC alpha power was analyzed relative to power in all the other frequencies, a 2 × 2 × 4 (measurement occasion by hemisphere by region) ANOVA revealed that *relative* EC alpha power showed a marginal decline from pre-task (T1: *M* = 1.92, *SE* = 0.84) to post-task (T2: *M* = 0.35, *SE* = 0.17), *F*(1,37) = 3.50, *p* < 0.07, $\eta_{p}^{2}$ = 0.09, with no other effects (*p*s > 0.25). Separate ANOVAs of EC power in the other frequencies indicated that like EC alpha power (*p* < 0.001, $\eta_{p}^{2}$ = 0.40), EC theta, beta, and gamma power increased following task performance (*p*s < 0.001,$\eta_{p}^{2}$ = 0.29–0.39), in contrast to delta power (*p >* 0.13, $\eta_{p}^{2}$ = 0.06), which did not change.
